# Our Censored Journals

**DOI:** 10.4103/0973-1229.39302

**Published:** 2008

**Authors:** David Healy

**Affiliations:** * *Professor of Psychiatry, Cardiff University, Wales LL57 2PW*

**Keywords:** *Data access*, *Fundamental attributional eerror*, *Ghostwriting*, *Legal suits*

## Abstract

When an article is rejected by a medical journal, the standard assumption is that the article is unsound or there is something wrong with the author. Alternatively, it may have been because the journal editor was concerned about the consequences should the article be published. This article seeks to inform discussion by providing a series of instances in which editorial concerns about the consequences to journals may have counted for more than any assessment about the truth-value of the article or the motives of its authors. This claim is based on the fact that different journals may treat exactly the same article in an entirely different fashion; some issues appear to be taboo in certain journals, no matter who the author, and there is a series of explicit communications from editors that publication has been held up by their legal departments.

## Introduction

Leemon McHenry's review of *Let Them Eat Prozac* in this journal (McHenry, 2007), with its accompanying editorial introduction and the author's account of the difficulties he faced in getting this review published, raises the question of self-censorship in medical journals (http://www.msmonographs.org/article.asp?issn=0973-1229;year=2007;volume=5;issue=1;spage=228;epage=232;aulast=McHenry).

The *New England Journal of Medicine* had originally accepted the review but, months later, changed their mind. The letter of acceptance was arguably a contract and McHenry might have considered suing for breach of academic freedom; instead he chose to send the review to the *Journal of Medical Ethics*, who accepted the review but whose legal advisors then counseled against publication for fear of libel, even though a review of the book had appeared in a sister journal, the *British Medical Journal.*

On publication of McHenry's review, the issue was taken up on the WAME listserve, where initial contributions focused on the qualities of the review and the book rather than on the dilemmas facing medical editors. This article outlines 14 sets of editorial decisions, in roughly historical order, that make it difficult to believe that the only factors involved in editorial decisions center on the article or idiosyncrasies of an author.

## Fourteen Editorial Decisions

### 1. A Mystery

In 1999, having agreed to testify medico-legally, I became aware of documents shedding light on the propensity of the selective serotonin reuptake inhibiting (SSRI) antidepressant Prozac (fluoxetine), to trigger suicidality and of the company's efforts to avoid giving warning of the risk. The documents were in the public domain but few were aware of their existence (Healy, 2004). My immediate thought was to write an article outlining the material for the *BMJ.*

When the question of Prozac-induced suicide was first raised in 1990, and the first legal actions had been filed against the company, the *BMJ* had carried an article with a company-only authorship line that, despite demonstrating a 1.9-fold increased risk of a suicidal act on Prozac compared to placebo, was widely spun as evidence that there was no risk from the treatment (Beasley *et al*., 1991). This article drew an intriguing response from a professor of psychiatry: “The *BMJ* is a journal of distinction and, dare I say it, perhaps also of some innocence. At a time when in the United States the manufacturer of fluoxetine is facing litigation, the corporate defense attorneys will be pleased by the journal having published a piece authored wholly by the manufacturer's employees”(Oswald, 1991).

Perhaps because of this criticism, the *BMJ'*s response to my submission was encouraging. The editor suggested reframing the article for the education and debate section of the journal. A revised article was sent to a reviewer, who was apparently not told that it was an education and debate article about company behaviour rather than an evidence-based assessment of the case for Prozac-induced suicidality. The reviewer suggested that the article had not established the case for treatment-induced problems - which, in fact, it had never attempted to do. The editor rejected the piece on this basis. Mystified at the mismatching messages, I appealed but in vain, with the editor in a phone call stating that no matter what revisions I made, nothing would be published. (All correspondence is available on www.healyprozac.com.) (Healy, 2004).

This article was published unaltered in the *International Journal of Risk and Safety in Medicine*, whose editor, Graham Dukes, commented that: “It seems to me your approach is original and fair… I have not seen the issues of litigation, regulation, and patents juxtaposed in this way before… I agree entirely from my own experience with many of your comments; there are some striking examples of companies tenaciously hanging on to a profitable and patented drug despite evidence that it is doing more harm than good. Their motives are a mixture of opportunism and genuine belief that the product is being wrongly accused. I also agree with your remarks about the failure of the present overall research approach to elicit a reliable picture of adverse effects and the sometimes unrealistic defenses put up by industry when their products are the subject of injury litigation” (quoted with permission). The article was given guest editorial status to emphasize its message (Healy, 1999a). A closely overlapping article appeared in the *Bulletin of Medical Ethics* (Healy 1999b).

### 2. Deepening Astonishment

A year later, having conducted a blind and randomized trial in healthy volunteers, in which two volunteers had become suicidal on an SSRI, I again contacted the *BMJ* about a submission but was told there was no point submitting the article. My assessment of the situation suggested seeking publication, instead, in a journal whose editors had previously worked within the pharmaceutical industry, on the basis that this background would make them less, rather than more, nervous about offending industry. The paper was reviewed and rapidly published (Healy, 2000a).

### 3. Financial Consequences

A further article published in the *Hastings Center Reports* (Healy, 2000b) made the case that most trials run by industry are marketing, rather than scientific, exercises; that articles, even in the best journals, are increasingly being ghostwritten; and that key data are suppressed. It transpired that Eli Lilly, the makers of Prozac, were the biggest single private donors to the Hastings Center. They withdrew their funding (Elliott and Chambers, 2004). The Hastings Center, in a possibly unprecedented step, sought to defuse the crisis by having the already published article re-reviewed. The re-review stated that all the points Healy made were valid - Healy's only problem was in not going far enough in criticizing industry practices (Elliott and Chambers, 2004).

### 4. When Nervous

Subsequently, I submitted a data-driven article to the *British Journal of Psychiatry* on ghostwriting, whose key finding was that a majority of the articles dealing with pharmaceutical products in our leading journals are likely to be ghostwritten. This journal usually has two peer reviewers. In this case the journal used at least five reviewers and had the revised article re-reviewed. The article was subsequently referred to the legal department of the journal and the copyeditors for the journal spent a great deal of time working on the final version (Healy and Cattell, 2003).

### 5, 6, 7, and 8. Fiduciary Responsibility?

Around this time, a much smaller journal *Contemporary Psychology* requested a review of Joseph Glenmullen's *Prozac Backlash* (2000). The review outlined the key points being made by the book, without endorsing the position of the author. It added that I was in possession of five highly critical reviews of the book by distinguished American psychiatrists, with accompanying documentation showing that public relations agencies working for Lilly had provided these reviews to media outlets and encouraged them not to feature the book. I sent the review and the accompanying documents to the editors. The review was initially accepted but failed to appear. On enquiring, I was told that the journal could not find a balancing reviewer and that they could not carry my review. The response made little sense.

When the issue of antidepressant-induced suicidality in paediatric populations emerged, *Open Minds* and *Young Mind* requested pieces on the issue. Both journals declined to publish on what I was told was legal advice. They made it clear that the decision was entirely because they had decided they did not have the resources to handle any difficulties they might run into with pharmaceutical companies as a result of the articles and that such difficulties could put them out of business (Available on request).

In 2005, the *Times Higher Education Supplement* (THES) featured a series of articles on Aubrey Blumsohn who had “blown the whistle” on Sheffield University and Proctor and Gamble over company concealment of data on the response to therapy with risedronate, a treatment for osteoporosis (THES, 2005). A series of letters were submitted to THES commenting on aspects of the case. Mine sought to make clear that Blumsohn's case was not unique. THES amendments to the letter stripped it of its meaning. I suggested that their revisions had made the letter pointless, to which they responded:“We have also had to run these letters past our lawyers as this is, as you are aware, a very sensitive issue and there are certain legal amendments we had to make” [Personal communication; available on request.]. They did not publish any letter from me.

### 9. Even Data is Tricky

The above articles largely involved commentaries. In 2004, *Evidence-Based Mental Health* approached me to provide a 300-word commentary on a *JAMA* article on antidepressants and suicide by Jick *et al.* (Jick *et al*., 2004). This article, which appeared in the middle of controversy as to whether the newer antidepressants might trigger suicidality in minors, appeared to exonerate these antidepressants of any risk. Following its publication, the FDA requested Dr. Jick to make available a further analysis that the published data obviously called for but which the manuscript did not include. This analysis suggested that the newer antidepressants were riskier than the older ones. My commentary put the new data from Dr. Jick in the public domain, with minimal additional comment. (This data is also available on www.fda.gov/ohrms/dockets/ac/04/transcripts/2004-4065T2.pdf (p154.)

This seemed to be an unusual development for the journal - frontline staff invoked the senior editors. In spite of indicating that it seemed to me that the best way forward surely was to have new evidence made available, perhaps with an accompanying comment by any other party of their choosing, the journal decided instead to abandon any comment on Dr. Jick's article.

In follow-up correspondence I noted that: “I think, looking at the confidence intervals in the originally published version, it was pretty clear that a reanalysis of the figures would throw up problems for anyone who was committed to the view that SSRIs pose no problems. And that's just what a reanalysis did.

“JAMA has also published another article on the treatment of adolescent depression (TADS) where again the abstract and headline and content are at variance with the data from the study which, by strict criteria, is a failed Prozac study. But JAMA have turned down pretty well all correspondence on the Jick article or the TADS article, while running lengthy commentaries praising these same articles, both of which have also attracted front page New York Times and Boston Globe coverage. At the same time, I and colleagues have sent a meta-analysis of all 677 (published) SSRI trials to JAMA, who have turned it down on the basis of a point that could have been handled by a simple rewording. Make what you will of this” (E-mail, DH to Sam Vincent of *Evidence-Based Mental Health;* 29/10/2004). The view of my colleague authors and I of this article was that the *JAMA* reviews had not pointed out any substantive problem with the article and, indeed, the *BMJ* later took the same article essentially unchanged and it has been among the top three cited articles in the *BMJ* in recent years.

### 10. The *BMJ* Revisited

In 2005, the *BMJ* had a new editor and I submitted an article on how the data on suicide and antidepressants had been manipulated. The peer reviews were longer than the original paper. After answering all queries, the paper was accepted. While in the middle of correcting the proofs, I received an e-mail from the editor: “Thank you very much for all your hard work on this article. I'm afraid we've run into a legal wall with our libel lawyer reluctant for us to publish your piece. I remain supportive of publication but obviously can't do this against legal advice.”

One consideration for the *BMJ* was that they were facing threats from Eli Lilly after running a news item about documents regarding the hazards of Prozac. Eventually, a year and a half later, possibly because of my persistence, the article was published (Healy, 2006a). The wording had been minimally altered to emphasize the failings of the regulatory authorities for the corrupted data in the public domain and to de-emphasize any failings on the part of the company.

### 11. Publishing Study 329

Study 329 was the key study of GlaxoSmithKline's (GSK) SSRI antidepressant, paroxetine, in depressed children. Faced with the results from this trial, company documents show GSK had concluded in 1998 that the drug did not work and that the data could not be presented publicly or even shown to the regulator. Nevertheless the “positive” aspects of the data would be selected for publication (see www.healthyskepticism.org/presentations/2007/Study329.ppt).

In 2001, an article reporting the results of 329 appeared in the *Journal of the American Academy of Child and Adolescent Psychiatry (JAACAP)*, the journal with the highest impact factor in child psychiatry. Apparently authored by some of the most distinguished psychopharmacologists in America, the article claimed that paroxetine was safe and effective for children (Keller *et al*., 2001). In fact, the paper was primarily authored by a medical writer. (E-mails from the company to the ghostwriter are available with this author.) The selected data and the claims presented in this paper were presented at a series of meetings by the “authors,” and sales of this drug, whose use in children was unlicensed, soared.

This is not the only case of its type, but the divide between what the published literature and the actual trial data show in the case of antidepressants given to children is possibly now the greatest known divide in all of medicine (Healy, 2006b). The processes that gave rise to this divide, however, can be reasonably assumed to apply to all other areas of therapeutics also.

The editors of our leading medical journals have attempted to clean up the mess posed by ghostwriting and lack of access to the underlying data from company studies by asking for authorship declarations and conflict of interest statements, rather than by requiring that companies make available the raw data. Asked baldly on BBC's investigative Panorama programme whether she would retract Study 329 or regretted its publication, now that it had been shown to be ghostwritten and misleading, the editor of *JAACAP* replied,“No” (Dulcan, 2007).

### 12. Critiquing Study 329

In 2007, I was approached by *Index on Censorship* for a piece outlining evidence “that pharmaceutical companies are not transparent and that medical journals allow this to happen. The implications of this for doctors and the general public would also have to be spelt out. You put it very succinctly when we spoke - pharmaceutical companies get to publish articles in major journals under the banner of science but they don't conform to the norms of science. The fact that there's this curious ‘gentleman's agreement,’ which means that pharmaceutical companies don't have to produce their data, should also of course be mentioned… I think, to an outsider who has certain expectations of science (that data is widely available and that access to data is fundamental in terms of any credibility), it's a baffling and shocking state of affairs” (E-mail; J. Glanville, Editor, *Index on Censorship* to DH, 20/05/2007).

The resulting article covered the evolution of ghostwriting and the lack of access to clinical trial data, focusing on Study 329. An iterative process began that finally got to the lawyers: “Our lawyer has just taken a look at your piece, and I do need to ask you for more chapter and verse on some points.

“I realize this is taking up more of your time than you bargained for and do apologize - lawyers must make you weary by now - but I am sure you'll understand that it's necessary.”

The process ended with: “The documents made interesting reading - and certainly answered the concerns - along with the cuts. But I've still got worries about running the piece. I regret how things have turned out very much. I've appreciated all your help in finding documents and in cooperating with all my requests. As I've said before - it's a hugely important subject and we should be covering it.” (Personal communication). *Index on Censorship* self-censored: the article is under review elsewhere.

### 13. Study 329 Re-critiqued

The difficulties with the publication of a critique of 329 do not seem to be solely due to its author. Following the emergence of evidence that SmithKline Beecham had viewed 329 as a failed study but nevertheless considered selecting the good bits for publication, the Lancet published an editorial (Editorial, 2004): “Depressing Research.” Subsequently, the journal published a letter from A. Benbow (Benbow, 2004) of GlaxoSmithKline claiming that the company was transparent on all issues to do with clinical trials. Leemon McHenry and Jon Jureidini wrote to the *Lancet* taking issue with Benbow's claims in a letter clearly stating that as an expert in the legal case involving Study 329 Jureidini had a conflict of interest. The *Lancet* agreed to publish their letter (letter available from author) but sent it first to GlaxoSmithKline who replied that it would not be appropriate to publish the letter, given Dr Jureidini's role as an expert witness involved in these issues, implying that seeking publication in the *Lancet* was a tactic designed to achieve a legal advantage (letter available from author). On this basis, the *Lancet* declined to publish McHenry and Jureidini's letter (letter available from author) even though the original Benbow letter could as readily be construed in this fashion, as New York State had taken a fraud action against GlaxoSmithKline for their lack of transparency in 329 and related studies, which the company later settled. (This charge and its settlement was widely reported by all major American media outlets; I was consulted as a medical expert by New York State.)

McHenry, Jureidini, and Peter Mansfield wrote a further paper on Study 329: “Clinical Trials and Drug Promotion: Selective Reporting in Study 329.” The editor of the *BMJ* wrote to them saying she had heard of their paper and wanted to fast track its publication. Six months later, after revisions, the *BMJ* indicated that their lawyers still had concerns and they would not publish.

### 14. The Other Side

In contrast to these difficulties in getting articles published, the process of publishing ghostwritten articles in major journals appears to be straightforward. In a 2006 *JAMA* editorial, Catherine de Angelis tackled the issue of why leading journals could not ban further articles from those linked to tainted articles, saying that “leveling sanctions against an author who fails to disclose financial interests by banning publication of his or her articles for some time period would only encourage that author to send his or her articles to another journal; it cleans our house by messing others. So what about all editors or at least a group, such as the ICMJE (International Committee of Medical Journal Editors), agreeing to share the information and jointly to ban the offending authors? Those who suggest this approach have not considered the risk of an antitrust suit” (De Angelis, 2006). This statement appears to concede that “scientific” journals cannot insist that contributors adhere to the norms of science by, for instance, being able to make publicly available the data on which their claims are based. This being the case, to avoid misleading a wider public, it might be better if publication outlets unwilling to commit to the norms of science were redesignated as periodicals rather than journals.

## Concluding Remarks

This selection of cases, on which the author has supporting documentation, points to editorial concern with issues other than the truth-value of articles submitted to journals. Are these isolated instances or have other articles of mine been turned down for similar reasons, with the rejection coated in terms other than the actual ones? Perhaps other commissioned reviews or articles have not come my way because of similar factors. Are other academics in a similar situation but unaware of, or unable to prove, the operation of factors of this sort?

Having been an object of interest for a number of public relations companies working for pharmaceutical companies, who have targeted me as a problem to be handled, I am aware of the things that can be said about the author in such cases. Editors are unlikely to be immune to open postings by pharmaceutical companies claiming that Healy “has distorted and mischaracterized the evidence… many erroneous statements, unsupported contentions, and data distortions… He has little scientific experience in conducting and interpreting the results of controlled clinical research… Before becoming a litigation expert witness testifying against SSRI manufacturers, Dr Healy published views opposite to those he now espouses on the question of whether SSRIs induce suicide” (Ryder, 2004).

Statements like this, which are actionably false, may have been made to invite a suit, knowing that such an action would drain a company critic of energy and time. The fact that I continue to leave statements like this uncontested may colour the attitude of editors to material that comes their way. The legal departments of journals will also necessarily take some cognizance of the fact that pharmaceutical companies actively explore the possibility of suing those they find inconvenient. I am in possession, through freedom of information requests, of documents from Eli Lilly indicating just such an approach (www.healyprozac.com/AcademicStalking/default.htm). Companies have the resources, and may have the incentive, to sue even if there is little prospect of winning.

In terms of the examples cited above, it might be possible to frame a great deal of what happened in terms of the personality and background of the author. There are hints however that this may not be all there is to it in that some journals have accepted an identical article to one other editors rejected, and other authors have had difficulties tackling the same issues. On the substantive issue - that antidepressants can trigger suicide - my position has been vindicated. Finally, the correspondence from journal editors indicates that the prospect of being sued is an issue for them.

In terms of the dynamics of how issues are portrayed, it is worth noting the existence of a fundamental attributional error (Kahneman *et al*., 1982). This is our tendency to expect that individuals are responsible for problems rather than to believe problems arise from the complexities of situations. Such a predisposition to look for villains may make it easier for medical editors to decide against an author rather than confront the difficulties of a situation.

At the end of the day, the cases above do seem to suggest that there is a contrast between journals’ difficulties in publishing material that is either entirely data driven or based on documents in the public domain, but which casts a company or drug in a bad light, and the apparent ease with which they accept articles that flout the central norms of science by refusing to permit access to the underlying data. It would be good to see some recognition that editors are faced with dilemmas in these areas - as without some recognition of this we are unlikely to generate solutions to the problem.

One possible helpful mechanism may be to have journals register articles submitted to them, just as companies now have to register clinical trials, with editors required to specify the reasons for non-acceptance of an article (quoted with permission; A. Blumsohn; personal communication). E-mails to and from the *BMJ* state: “The *BMJ* Group is one of the world's most trusted providers of medical information for doctors, researchers, health care workers, and patients.” But the review process at *BMJ* and other journals is not transparent. Opening the process up to scrutiny might bolster trust.

Some of the issues raised here might be defused if the data from clinical trials were open to scrutiny. At present, journals permit companies to publish material without requiring that they conform to the norms of science by making the data available. These articles have become a primary tool for companies, who use them to market compounds under the banner of science. Availability of data might enable journals to publish defensible alternate claims.

### Take Home Message

Many articles on medical issues interface with the business of medicine as much as its science base and, as such, the publication or non-publication of these articles may hinge not only on the truth-value of the article's contents or the qualities of its author but also on an editor's perception of the problems a company may pose to the journal.

## Questions That This Paper Raises

1. Why are journals apparently unwilling to ensure that publications relating to pharmaceutical products conform to the norms of science in making data available?
2. Why are academic meetings unwilling to ensure that presentations of pharmaceutical company data conform to the norms of science by making data available?
3. Do our medical publications still deserve the sobriquet journals or should they be renamed periodicals?
4. Is it time to rebrand our academic meetings as trade fairs? Even if not entirely given over to marketing, the presence of non-company presentations and material at such meetings helps generate an impression of science that is useful for marketers.
5. Would the marketing departments of pharmaceutical companies prefer the public at large to think that the real issues center on undeclared conflicts of interest or on the failures of journal editors and academic meeting organizers to ensure that journals and meetings are in fact scientific?

## About the Author

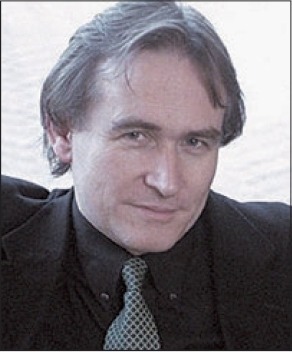



David Healy is a Professor of Psychiatry in Cardiff University, a former Secretary of the British Association for Psychopharmacology, and author of over 150 peer reviewed articles, 200 other pieces, and 15 books, including The Antidepressant Era and The Creation of Psychopharmacology from Harvard University Press, The Psychopharmacologists Volumes 1-3, and Let Them Eat Prozac from New York University Press, with Mania forthcoming from Johns Hopkins University Press.

He has been involved as an expert witness in homicide and suicide trials involving SSRI drugs and in bringing these problems to the attention of American and British regulators. He has raised awareness of how pharmaceutical companies market drugs by marketing diseases and co-opt academic opinion leaders by ghostwriting their articles.
